# The *yhiM* gene codes for an inner membrane protein involved in GABA export in *Escherichia coli*

**DOI:** 10.3934/microbiol.2017.1.71

**Published:** 2017-02-17

**Authors:** Angela Tramonti, Fiorenzo De Santis, Eugenia Pennacchietti, Daniela De Biase

**Affiliations:** 1 Institute of Molecular Biology and Pathology, CNR, Department of Biochemical Sciences “A. Rossi Fanelli”, Sapienza University of Rome, Piazzale Aldo Moro 5, 00185 Roma, Italy; 2 Department of medico-surgical Sciences and Biotechnologies, Sapienza University of Rome, Laboratory affiliated to Istituto Pasteur Italia-Fondazione Cenci Bolognetti, Corso della Repubblica 79, 04100 Latina, Italy

**Keywords:** inner membrane proteins, acid resistance, *Escherichia coli*, GadX, GadE, anaerobiosis

## Abstract

In order to survive the exposure to acid pH, *Escherichia coli* activates molecular circuits leading from acid tolerance to extreme acid resistance (AR). The activation of the different circuits involves several global and specific regulators affecting the expression of membrane, periplasmic and cytosolic proteins acting at different levels to dampen the harmful consequences of the uncontrolled entry of protons intracellularly. Many genes coding for the structural components of the AR circuits (protecting from pH ≤ 2.5) and their specific transcriptional regulators cluster in a genomic region named AFI (acid fitness island) and respond in the same way to global regulators (such as RpoS and H-NS) as well as to anaerobiosis, alkaline, cold and respiratory stresses, in addition to the acid stress. Notably some genes coding for structural components of AR, though similarly regulated, are non-AFI localised. Amongst these the *gadBC* operon, coding for the major structural components of the glutamate-based AR system, and the *ybaS* gene, coding for a glutaminase required for the glutamine-based AR system. The *yhiM* gene, a non-AFI gene, appears to belong to this group. We mapped the transcription start of the 1.1 kb monocistronic *yhiM* transcript: it is an adenine residue located 22 nt upstream a GTG start codon. By real-time PCR we show that GadE and GadX equally affect the expression of *yhiM* under oxidative growth conditions. While YhiM is partially involved in the RpoS-dependent AR, we failed to detect a significant involvement in the glutamate- or glutamine-dependent AR at pH ≤ 2.5. However, when grown in EG at pH 5.0, the *yhiM* mutant displays impaired GABA export, whereas when YhiM is overexpressed, an increases of GABA export in EG medium in the pH range 2.5–5.5 is observed. Our data suggest that YhiM is a GABA transporter with a physiological role more relevant at mildly acidic pH, but not a key component of AR at pH < 2.5.

## 1. Introduction

The ability to sense and respond to a mild-to-harsh acid stress (from pH 6.0 down to pH 2.0) is essential for neutralophilic bacteria, such as *Escherichia coli*, because inorganic and organic acid stresses encountered in the external environment, but also in host districts such as the gastric and distal gut compartments as well as the macrophage phagolysosome, can lead to bacterial death [1],[2]. Several studies have provided insight into the environmental and regulatory factors affecting the expression of the genes belonging to the most potent acid resistance (AR) system in the enteric bacterium *Escherichia coli* K12, namely the glutamate-dependent AR (GDAR or AR2) system (For reviews: [1],[3]). In order to function the *E. coli* GDAR system requires glutamate supplementation in the pH 2.5 minimal medium used for acid challenge [4], the intracellular proton-consuming activity of at least one of the two isoforms of glutamate decarboxylase (GadA or GadB) and the antiport activity of GadC, an inner membrane protein which exchanges exogenous glutamate for γ-aminobutyrate (GABA), the decarboxylation product [5],[6].

**Figure 1.**
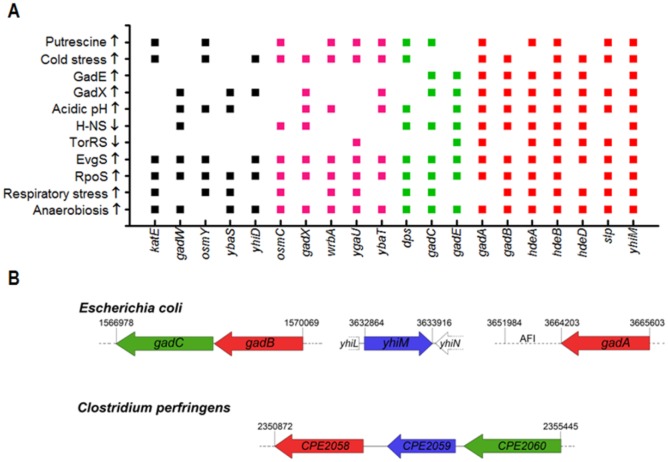
Preliminary observations suggesting that *yhiM* might belong to the AR circuit. **(A)** In the graph the genes found in 5–6 (black squares), 7 (magenta squares), 8 (green squares), 9–11 (red squares) out of the 11 transcriptomic studies taken under consideration [16],[17],[19]–[23],[25],[26],[28],[29],[30] are listed for each group in alphabetical order. The environmental stimuli and the regulators analysed in each transcriptomic study are listed on the Y axis and arrows show up-regulation (↑) or down-regulation (↓) of the reported genes. **(B)** Schematic representation of the genomic regions containing the *gadB, gadC, gadA* and *yhiM* genes in *E. coli* K12 MG1655 and its homologues in *Clostridium perfringens* str.13. Orthologs and paralogs are depicted in the same colour for both microorganisms. The 5′ end of the *E. coli* AFI is indicated.

Acid stress, starvation, hyperosmolar stress, anaerobiosis, cold and respiratory stress were shown to cause significant up-regulation of most of GDAR genes (Figure 1A and references therein). The activation of these genes is essentially controlled via two circuits which function under different growth conditions: the cross-talking phosphorelay EvgSA and PhoQP circuits become operative when exponentially growing cells are cultivated in minimal medium at mildly acidic pH and under a low Mg^2+^ concentration, respectively, whereas the GadXYW circuit plays a major role when cells grown in rich medium enter into the stationary phase [7],[8],[9]. Both circuits converge on the activation of the regulator GadE, the role of which is essential to fully develop GDAR [10], as also reported for pathogenic *E. coli* [3]. The genes involved in GDAR encode in addition to the two paralogs of glutamate decarboxylase (*gadA* and *gadB*) and the glutamate/GABA antiporter (*gadC*), also two periplasmic chaperons (*hdeA* and *hdeB*), and specific transcriptional regulators belonging either to the AraC-family (*gadX*, *gadW, ydeO)* or to the LuxR-family (*gadE* and *yhiF*). Though less extensively investigated, the protein products of the *slp*, *yhiD* and *hdeD* genes also contribute to AR [11]. With the notable exception of *ydeO* and *gadBC*, all the above listed genes are clustered at approximately 3.66 Mb on the *E. coli* K12 chromosome, in the acid fitness island (AFI) [12]. The gene *ybaS* (*glsA*) coding for a glutaminase is also distantly located from AFI and *gadBC*, and plays a key role in the glutamine-dependent AR system (AR2_Q), which also requires GadC for the entry of glutamine and the export of glutamate or GABA [1],[13],[14],[15]. Besides the activating circuits, the TorSR phosphorelay system was shown to negatively affect GDAR genes expression under alkaline conditions [16]. As part of the complex network controlling the expression of these AR genes, RpoS, the general stress sigma factor of the RNA polymerase, and H-NS, a global transcriptional regulator acting mostly as gene silencer, are both playing an important role as they positively and negatively influence the expression of the above genes, respectively [17],[18].

Notably, in several transcriptomic studies in addition to *ybaS*, *yhiM*, another non-AFI gene, is frequently found in the list of genes regulated alike many AR genes (Figure 1A). In particular *yhiM* is activated under acidic, cold and respiratory stresses as well as in anaerobic conditions, repressed by H-NS and TorSR, in addition to being RpoS-dependent and activated by EvgAS, GadE and GadX [16],[18],[19]–[26]. In this report we analyse in further detail the transcriptional regulation of the *yhiM* gene in the *E. coli* strains MC4100 and MG1655, a widespreadly used strain and a reference K12 strain for acid resistance studies, respectively. Respect to what was suggested by other authors using a less-characterized *E. coli* strain UCB [27], in MC4100 and MG1655 we observed only a partial involvement of the inner membrane protein YhiM in the RpoS-dependent AR system, but did not observe a clear involvement in the glutamate- or glutamine-dependent AR at pH 2.5. On the other hand, we observed that YhiM affects GABA export at pH 5.0 in minimal medium.

## 2. Materials and Methods

### 2.1. Bacterial strains, plasmids and growth media

The bacterial strains and the plasmids used in this study are listed in Table 1. Bacterial growth was monitored by determining the optical density at 600 nm (OD_600_) with a diode array spectrophotometer (HP8452; Agilent Technologies). The media used were: LB [31], LB-MES (LB buffered at pH 5.5 with 100 mM morpholineethanesulfonic acid [MES]) or brought to pH 2.5, LB-MOPS (LB buffered at pH 7.4 with 100 mM 3-[N-Morpholino]propanesulfonic acid [MOPS]) and EG, minimal medium E [32] supplemented with 0.4% glucose, at the desired pH. Antibiotics were added at the following concentrations: ampicillin, 100 µg/ml; streptomycin, 20 µg/ml; kanamycin, 25 µg/ml. The Δ*yhiM* and Δ*gadC* deletion mutants from the *E. coli* K12 strains MG1655 and MC4100 were generated using the procedure described by Datsenko and Wanner [33] with the oligonucleotides pairs Δ*yhiM_*for-Δ*yhiM_*rev and Δ*gadC_*for-Δ*gadC_*rev, respectively (Table 2). The replacement of the *yhiM* (or *gadC*) ORF with a Kan^R^ cassette, following a double crossing-over on the target gene, was checked by comparing the size of the PCR products obtained by amplifying the genomic DNA of the wild type and the mutant strains with pairs of primers annealing upstream and downstream of the deleted ORFs *yhiM_*for/*yhiM (Bgl)*_rev, for the *yhiM* mutant; α/β_out/*gadC*_rev, for the *gadC* mutant (Table 2).The insertion of the Kan^R^ cassette was checked with oligo k1 (specific for the cassette) and *yhiM (Bgl)*_rev, for the *yhiM* mutant or *gadC*_rev, for the *gadC* mutant (Table 2). Both mutants did not show any growth defect in LB and EG media at neutral and mildly acidic pH, when growth was carried out at 37 °C.

### 2.2. RNA analysis

RNA was isolated from cells grown under the specified conditions, using either a modified hot-phenol extraction method [5] or the RNeasy mini kit (QIAGEN). RNA concentration and quality were assessed by determining (in 0.1 N NaOH) the optical density at 260 nm (OD_260_) and the 260 nm/280 nm ratio, respectively. Electrophoresis was carried out essentially as previously described [5] loading 5 µg of total RNA per sample. To detect *yhiM*-specific mRNA, northern blots were hybridized with a 1050-bp *yhiM* probe obtained by PCR amplification using plasmid pCRII*yhiM* (see below) as template and the oligonucleotide pair *yhiM_*for and *yhiM (Bgl)*_rev (Table 2). The *yhiM*-specific probe was labelled and hybridized with the DIG High Prime Labeling and Detection Starter Kit II (Roche), according to manufacturer instructions.

Primer extension analysis was performed with the oligonucleotide *yhiM_*revRT (Table 2), which anneals 286 nt downstream the putative GTG start codon, and using total RNA (7.0 µg) extracted from either *E. coli* MC4100 or *E. coli* MG1655 grown to the stationary phase in LB-MES, pH 5.5. The entire procedure was essentially as previously described [5].

**Table 1.** Bacterial strains and plasmids used in this study.

Strain or Plasmid	Relevant Genotype	Reference or source
MC4100	F^−^ *araD139* Δ*(argF-lac)U169 rpsL150 relA1 flbB5301 deoC ptsF25 rbsR*	[34]
MG1655	F^−^ λ^−^ *rph*^−*1*^	GSC
JM109	(F' *traD36 proA^+^ proB^+^ lacI^q^ lacZ*Δ*M15) recA1 endA1 gyrA96 thi hsdR17 supE44 relA1* Δ(*lac-proAB*)	[31]
MC4100*gadX*	MC4100 *gadX*::Kan^R^	[9]
MC4100*gadE*	MC4100 *gadE*::Kan^R^	[35]
MG1655*gadX*	MG1655 *gadX*::Kan^R^	[9]
MG1655*gadE*	MG1655 *gadE*::Kan^R^	[35]
MG1655*yhiM*	MG1655 *yhiM*::Kan^R^	This work
MC4100*yhiM*	MC4100 *yhiM*::Kan^R^	This work
MG1655*gadC*	MG1655 *gadC*::Kan^R^	[36]
MC4100*gadC*	MC4100 *gadC*::Kan^R^	This work
pBs	(pBluescriptSK) multicopy phagemid vector; ColE1 replicon, *lacZ* *bla*	Stratagene
pCRII*yhiM*	1050-bp fragment encompassing the entire *yhiM* coding sequence ligated in pCRII-TOPO	This work
pQE60	Expression vector; ColE1 replicon, P_T5_-*lacO* RBSII	QIAGEN
pQE*yhiM-*6×His	1050-bp fragment encompassing the entire *yhiM* coding sequence ligated to the *Nco*I-*Bgl*II sites of pQE60	This work

### 2.3. RT-qPCR

Quantitative real time RT-PCR (RT-qPCR) reactions were performed in two steps. Reverse transcription of DNase-treated RNAs (3 µg) was carried out using 10 U Transcriptor Reverse Transcriptase (Roche) and oligonucleotides *rRNA16S_*rev, and *yhiM_*revRT (Table 2). Real Time PCR was performed on a Chromo4 Real Time PCR Instrument (BioRad Laboratories) using the LightCycler FastStart DNA Master SYBR Green I (Roche) and the following pairs of oligonuleotides (Table 2): *yhiM_*for-*yhiM_*revRT for amplification of *yhiM*-specific mRNA, and *rRNA16S_*for-*rRNA16S_*rev for amplification of the 16S rRNA (internal control).

### 2.4. Cloning of yhiM

A 1050-bp DNA fragment encompassing the entire *yhiM* open reading frame was generated by PCR using Fast Start High Fidelity Enzyme (Roche) and the *E. coli* MG1655 genomic DNA as template, with the oligonucleotides *yhiM_*for and *yhiM (Bgl)*_rev (Table 2). The amplicon was subcloned into pCRII-TOPO, using the TOPO TA Cloning System (Invitrogen) and sequenced on both strands. The *Nco*I-*Bgl*II fragment from pCRII*yhiM* was then ligated to the corresponding sites of the expression vector pQE60, linearized with the same restriction enzymes. In the newly generated plasmid construct, named pQE*yhiM-*6×His, the *yhiM* gene is cloned in frame at its 3′ end with the nucleotide sequence coding for a 6×His tag carried by pQE60. This plasmid pQE*yhiM-*6×His was used to transform competent cells of *E. coli* JM109/pREP4.

To assay YhiM-directed GABA export, single colonies of the *E. coli* strains JM109/pREP4/pQE60 and JM109/pREP4/pQE*yhiM-*6×His were inoculated in 3 ml of LB, containing ampicillin and kanamycin, grown to the late exponential phase and then induced by addition of 0.2 mM IPTG. Growth was prolonged overnight, after which cultures were centrifuged at 3500 rpm for 10 min and the pellet resuspended in 1 ml EG, pH 7.0. The washing and resuspension steps were repeated one more time. Aliquots of 25 µl were transferred in 475 µl of EG medium, at different pH (2.5, 3.5, 4.5 and 5.5), containing 1 mM sodium glutamate. Incubation was carried out for 90 min at 37 °C and the reactions were stopped and neutralized by adding 4 µl of 5 N NaOH. Following a centrifugation step to pellet the bacteria, 50 µl of the reaction mixtures were assayed with the Gabase assay, as previously described [37]. Cell viability was checked by plating out the bacteria before and after the challenge at the indicated pH.

**Table 2.** Oligonucleotides used in this study.

Primer	Sequence		Restriction site
Δ*yhiM*_for	5′-ATTATCATTAATGCATATTTCAATATTAGCAGGGATACC gtgtaggctggagctgcttc-3′		
Δ*yhiM*_rev	5′-CCGCTTCTAATATTGAAACGATTGAGAACAACGTAAAGC attccggggatccgtcgacc-3′		
Δ*gadC*_for	5′-TACCGTTTTAGGGGGATAATATGGCTACATCAG TACAGACgtgtaggctggagctgcttc-3′		
Δ*gadC*_rev	5′-TTAGTGTTTCTTGTCATTCATCACAATATAGTGTGGTGAA attccggggatccgtcgacc-3′		
*yhiM*_revRT	5′-TAACAGTGCCCAACCCCATATCAT-3′		
*yhiM_*for	5′-ggataccatgGTGAACATATATATCGGGTGG-3′		NcoI
*yhiM (Bgl)*_rev	5′-ggttagatctTTTTTTAGCAGAACCCGCTTC-3′		BglII
*gadC*_rev	5′-GCACATATGGATATCTGCTCCC-3′		
α/β_out	5′-CGAAACTGCAGGGTATTGC-3′		
*rRNA16S_*for	5′-CGTTACCCGCAGAAGAAGC-3′		
*rRNA16S_*rev	5′-GTGGACTACCAGGGTATCTAATCC-3′		
k1	5′-CAGTCATAGCCGAATAGCCT-3′		

Restriction sites are underlined. Nucleotides not matching the target sequence are in lower-case.

### 2.5. Cell fractionation

Cytoplasmic and membrane fractions from *E. coli* wild-type strain MC4100 and its mutant derivative MC4100Δ*yhiM* were obtained as described by Capitani et al. [38] with some minor modifications. Briefly, after 24-hours growth in LB, the bacterial pellet from a 0.5 L culture was resuspended in 25 ml of 50 mM Tris-HCl, pH 7.5, containing 1 mM DTT and a protease inhibitor cocktail (Complete, Roche). The resuspension was then divided into two 13-ml aliquots. One of the aliquots was brought to pH 5.6 by dropwise addition of 8 N HCl. The two samples were then sonicated and if necessary the pH was adjusted to 7.4 and 5.6, respectively. An additional slow speed centrifugation step was carried out to remove cell debris and unbroken cells, thus generating the cell extract. The cytoplasmic and membrane fractions from each cell extract were separated by ultracentrifugation at 50,000 rpm (Beckman model L8-70) for 1 h at 10 °C. The pellet (corresponding to the membrane fraction) was resuspended in 2 ml of 100 mM Tris-HCl, pH 8.0, containing 150 mM NaCl, 5 mM EDTA and 0.5% lauroyl sarcosine.

### 2.6. AR assays

Oxidative and fermentative AR systems were assayed essentially as described by Lin et al. [4]. Briefly, 24-hours cultures in either LBG, pH 5.0 (for fermentative AR) or LB-MES, pH 5.5 (for oxidative AR) were acid challenged by 1:1000 dilution in rich medium at pH 2.5 or minimal medium EG at pH ≤ 2.5, supplemented when required with the relevant amino acids to test for the fermentative amino acid-dependent AR systems.

### 2.7. Gad activity assay and GABA measurements

Glutamate decarboxylase activity assays and GABA measurements were performed as previously described [37].

## 3. Results and Discussion

### 3.1. Transcriptional regulation of yhiM

As mentioned in the Introduction, in several transcriptomic studies the *E. coli* *yhiM* gene, a non-AFI gene, is frequently found in the list of genes regulated alike many AR genes (Figure 1A). In the *E. coli* K12 reference strain MG1655 the *yhiM* gene (b3491) is located in between the divergently oriented *yhiL* and *yhiN* genes (Figure 1B), thus suggesting that its transcript is monocistronic. The genomic context in *E. coli* is rather different from that found in *Clostridium perfringens* str.13, where the gene *CPE2059* coding for a 351 amino acid-long protein homologous to *E. coli* YhiM, is located in between the genes *CPE2060* and *CPE2058*, coding for the homologs of *E. coli* GadC and GadB, respectively (Figure 1B and [12]). A similar gene assembly is also present in other strains of *C. perfringens* for which the genome sequence is available (data not shown).

These preliminary observations, i.e. co-regulation with AR genes (Figure 1A) and co-localization with the genes coding for GadB and GadC in *C. perfringens* (Figure 1B), prompted us to further investigate both the transcriptional regulation of *E. coli* *yhiM* and the biological role played by its protein product, in light of a possible involvement in AR and specifically in GDAR. As a further reason of interest, *yhiM* codes for an integral inner membrane protein belonging to a protein family, DUF2776, the function of which is not known.

**Figure 2.**
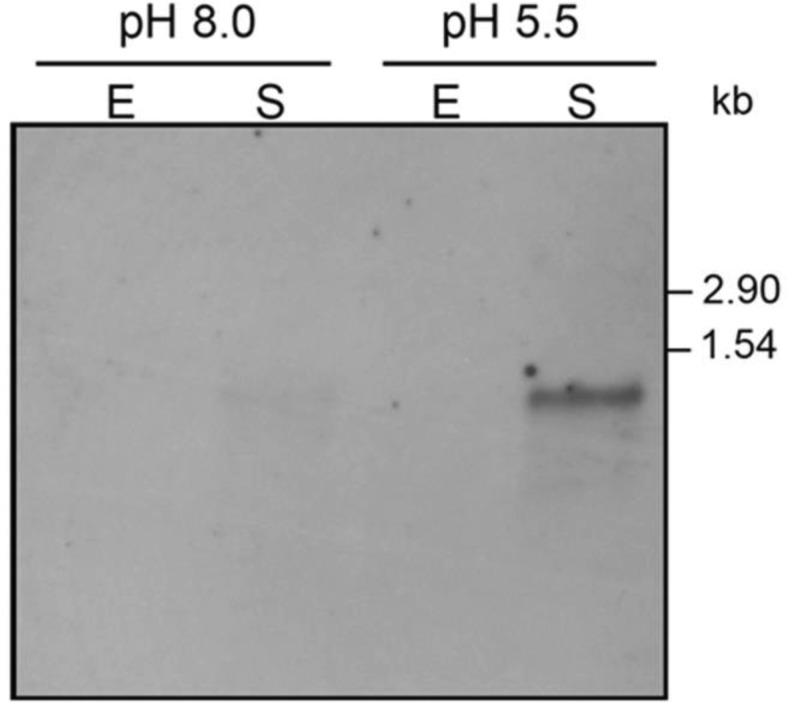
Northern blot analysis of *yhiM*-specific transcript. Total RNA was extracted from *E. coli* strain MG1655 grown to the exponential (E, OD_600_ ∼ 0.6) and stationary (S, OD_600_ ∼ 2) phases, in LB-MOPS (pH 8.0) or LB-MES (pH 5.5).

To study *yhiM* transcriptional regulation, total RNA was extracted from *E. coli* K12 MG1655 cells grown to the exponential or stationary phases either in LB-MOPS medium (pH 7.4) or in LB-MES medium (pH 5.5). Northern blots of total RNA (5 µg/sample), resolved on 1.2% agarose gels, were hybridised with a DIG-labelled *yhiM*-specific probe. Figure 2 shows that the *yhiM* probe detects a single mRNA species with an apparent length of 1.1 kb, as deduced from its electrophoretic mobility. The *yhiM* mRNA is detectable only in the stationary phase samples, particularly in that obtained from bacteria grown at pH 5.5 under oxidative conditions, i.e. LB-MES pH 5.5. Similar results were obtained when RNA extracted from *E. coli* MC4100 was analysed by Northern blot (data not shown).

The *yhiM* transcription was then analysed quantitatively by real time PCR in *E. coli* MG1655 and in its isogenic *gadX* and *gadE* derivatives grown in different media and conditions (Table 3). We found that the *yhiM* transcript is 9 folds more abundant at pH 7.4 in exponential phase under anaerobic conditions and 12.5 folds more abundant in the stationary phase of growth at pH 5.5 with respect to the exponential phase under aerobic conditions. In the *gadX* and *gadE* mutants, analysed under the most inducing condition (i.e. stationary phase in LB-MES pH 5.5), *yhiM* expression is still higher than in the stationary phase at pH 7.4, but nevertheless reduced by half with respect to that observed in the wild-type strain at pH 5.5 (Table 3, last three columns).

**Table 3.** Expression fold change of the *yhiM*-specific transcript in *E. coli* MG1655 in different conditions and mutant backgrounds as assessed by quantitative real time PCR.

Strain	MG1655				Δ*gadX*	Δ*gadE*
Medium	*LB-MOPS pH 7.4*			*LB-MES pH 5.5*		
Condition	*E-O_2_*	*E*	*S*	*S*		
**Expr. fold**	9	1	1.5	12.5	5.7	6
**SEM**	3		0.08	1.5	1.1	1.2
**P value**	0.0001		0.01	0.002	0.002	0.006

Total RNA was extracted from *E. coli* K12 strain MG1655 and its *ΔgadX* and Δ*gadE* derivatives grown to the exponential aerobic (E), exponential anerobic (E-O_2_) and stationary (S) phases, in the indicated medium.

**Figure 3.**
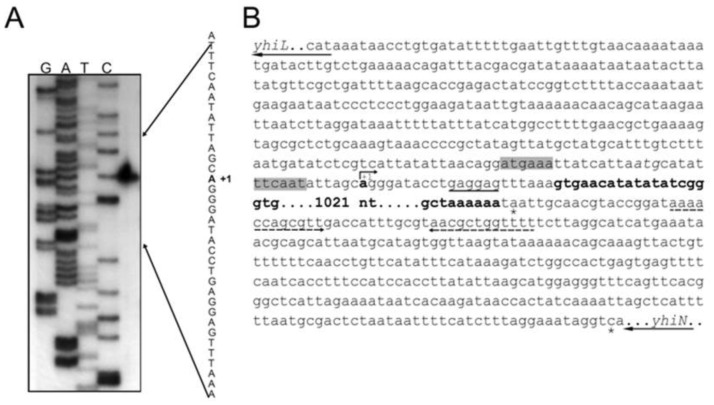
Sequence analysis and mapping of the *yhiM* transcriptional start site. **(A)** Mapping of the 5′' end of the *yhiM* transcript by primer extension analysis. RNA was extracted from *E. coli* MC4100 stationary phase cells grown at pH 5.5 and reverse transcribed after priming with the oligonucleotide *yhiM*_revRT (Table 2), labelled at its 5′-end. Lanes G, A, T, and C are sequencing ladders of pCRII*yhiM* with the same oligonucleotide used for the primer extension reaction. Sequencing reactions were run in parallel with the cDNA transcript (right lane). **(B)** Nucleotide sequence of the *yhiL*-*yhiN* genome region in *E. coli* MG1655. The bent arrow indicates the *yhiM* transcriptional start site at the residue in bold (+1). The sequences for the –10 and –35 putative promoter elements are on gray background. The potential Shine-Dalgarno sequence for ribosome binding is underlined. The *yhiM* ORF is in bold. The asterisks indicate the *yhiM* and *yhiN* stop codons. The dashed arrows indicate the inverted repeats probably generating a stem-loop like structure. The black arrows at the 5′ and 3′ ends of the sequence show the start and stop triplets as well as the direction of transcription of the *yhiL* and *yhiN* genes, respectively.

In order to localize more precisely the –10 and –35 promoter elements (also in light of more in depth transcription regulation studies) and to drive the design of primers correctly placed for mutant construction, primer extension analysis was conducted to identify the 5′-terminus of the *yhiM* mRNA using total RNA extracted from *E. coli* MC4100 grown to the stationary phase in LB-MES, pH 5.5. Results reported in Figure 3A show that transcription of *yhiM* originates from an adenine (A) residue located 22 nt upstream a GTG codon, which functions as a translation start in *E. coli* [40]. An identical result was obtained when total RNA from *E. coli* MG1655 was used instead (data not shown). At compatible distance from the 5′ end of the *yhiM* transcript, we identified –35 (ATGAAA) and –10 (TTCAAT) promoter elements, optimally spaced by 17 nt (Figure 3B). Both –10 and –35 sequences match 4 out of 6 nucleotides of the recognition sites for σ^70^-dependent RNA polymerase. Moreover, the sequence GAGGAG-N_6_, immediately upstream the GTG start codon, is fully compatible with the Shine-Dalgarno sequence for ribosome binding (Figure 3B). The 1127-nt length of the *yhiM* transcript, deduced from the distance between the experimentally determined transcription start site and a putative stem-loop structure centred 35 nt downstream the *yhiM* stop codon (Figure 3B), perfectly agrees with the predicted length of the transcript as deduced by Northern blot analysis (Figure 2).

### 3.2. YhiM does not affect GadA/B and GadC cellular localization and is not a key component of GDAR

*E. coli* YhiM, starting from the methionine residue coded by the GTG alternative start codon, is a 350 amino acid-long protein with a calculated molecular mass of 37.66 kDa and a theoretical pI 8.2. A BLAST [41] search, using *E. coli* YhiM (Swiss-Prot: P37630) as query sequence, retrieved as best scoring sequences (>45% identity) those from members of a protein family (pfam10951: DUF2776) for which a biochemical function has not been assigned yet. The Clustal Omega alignment of *E. coli* YhiM and its homologues from *Bordetella avium*, *Laribacter hongkongensis*, *C. perfringens*, *Bacteroides tethaiotaomicron* and *Parabacteroides distasonis* is provided in the Supplementary Figure 1. Based on several topology prediction programs (www.expasy.org), *E. coli* YhiM consists of 10 transmembrane (TM) spanning domains, with both the N-terminal and C-terminal ends on the cytoplasmic side. The amino acids predicted in TM domains are indicated by black lines above the alignment in Supplementary Figure 1. Overall the alignment shows that the degree of identity at the level of the predicted TM domains (35% on average) is that mostly contributing to the overall identity.

In order to answer the question on the role played by YhiM, we first investigated a possible involvement in the physical and/or functional interaction with GadA/B or GadC. In fact, one possibility is that YhiM may provide the membrane anchoring site for GadB when it is recruited to the membrane district upon acidification of the cytoplasm [38]. Indeed, it was shown that in the cell extracts from the *E. coli* strain JM109 overexpressing GadB, the enzyme at neutral pH is mainly localized in the cytoplasmic fraction, whereas at mildly acidic pH (pH 5.6) more than 55% of the protein is detected and assayed in the detergent-solubilized membrane fraction [38]. To date the chemical nature (protein or lipid) of the anchoring partner is still not known. Experiments carried out in our laboratory suggest that recruitment of GadB to the membrane does not occur *via* GadC, the cognate membrane antiporter (data not shown) in GDAR. In order to investigate whether YhiM could be involved in the pH-dependent recruitment of glutamate decarboxylase to the membrane, both the cytosolic and membrane fractions from the *E. coli* strain MC4100 and its *ΔyhiM* derivative were assayed for Gad activity both at pH 7.4 and pH 5.6. The results are shown in Table 4 and provide evidence that, as in the overexpressing strain [38], in the wild-type strain at acidic pH native glutamate decarboxylase (consisting of a mixture of GadA and GadB; [37]) partitions between the cytosolic and membrane fractions and that in the absence of *yhiM* no major changes are observed. According to these results, we can exclude that YhiM is the protein partner recruiting GadA/B to the membrane and also exclude that the *yhiM* mutation affects the GadA/B activity in the cell. In fact the total enzyme units in wild type MC4100 (300 Units/OD_600_) are very close to those measured in the Δ*yhiM* mutant (330 Units/OD_600_).

**Table 4.** Partition of Glutamate decarboxylase (GadA/B) activity* in the cytosolic (Cyto) and membrane (Mem) fractions from stationary phase cellular extract brought to pH 7.4 or 5.6.

	pH 7.4		pH 5.6	
	*Cyto*	*Mem*	*Cyto*	*Mem*
**MC4100**	265 ± 21	75 ± 6	146 ± 11	124 ± 9
**MC4100Δ*yhiM***	275 ± 23	56 ± 4	190 ± 8	143 ± 5

* the activity is reported in Units/mg.

Being a membrane protein, an alternative role played by YhiM could be that of participating to GDAR either by physically interacting with GadC or by functionally overlapping its function. Therefore we analysed the effect of *yhiM* deletion on both the GadC localization in the cell membrane and the involvement in the GDAR phenotype.

Immunoblot analysis of total cell extracts and membrane fractions from the *E. coli* wild-type strain MC4100 and its Δ*yhiM* derivative, using anti-GadC antibodies, showed that GadC expression and localization in the cell membrane are not altered in the absence of YhiM (data not shown).

**Figure 4.**
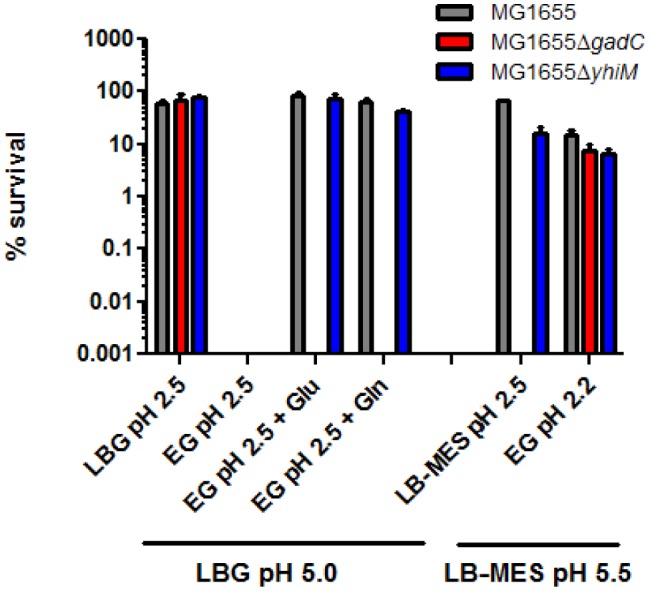
Participation of *E. coli* *yhiM* to fermentative and oxidative AR systems. Wild type MG1655 and the isogenic *gadC* and *yhiM* mutants were tested for different AR phenotypes. G glutamate- and glutamine-dependent AR were assayed by diluting 1:1000 24-hours cultures grown in LBG pH 5.0 in the indicated media. The oxidative AR system activity was assayed by diluting 1:1000 24-hours cultures grown in LB-MES pH 5.5 in the indicated media.

Also GDAR was not affected in the absence of a functional *yhiM* gene. Using the standard conditions to assay the RpoS-, glutamate- and glutamine-dependent AR phenotypes, we analysed both the Δ*yhiM* and the Δ*gadC* strains and compared their AR phenotype with that of wild-type MG1655. The results (Figure 4) are in disagreement with the data provided by Nguyen and Sparks-Thissen [27] as we failed to observe any major involvement of *yhiM* in acid resistance, with only the RpoS-dependent circuit being slightly affected. On the contrary the *gadC* mutation is sufficient to give rise to an acid sensitive phenotype, as expected from previous studies [5],[6].

It has been reported that MG1655 survives extremely well in pH 2.5 spent EG medium, whereas it succumbs in fresh EG at pH 2.5, in the absence of glutamate. A likely explanation for this observation is that glutamate secretion, which is proposed to originate by metabolic overflow [42], would provide sufficient substrate in the spent medium to support GDAR, even in the absence of glutamate supplementation. We therefore assayed GDAR in spent EG medium, acidified to pH 2.5, obtained from either *E. coli* MG1655 or MG1655Δ*yhiM* grown for 14–15 hours. Wild type MG1655 survived well in both acidified spent challenge media (data not shown). This suggests that the Δ*yhiM* strain is not impaired in the ability to export glutamate (or any other molecule) required to protect against acid challenge in spent EG medium.

### 3.3. YhiM participates to GABA export in E. coli at acidic pH

**Figure 5.**
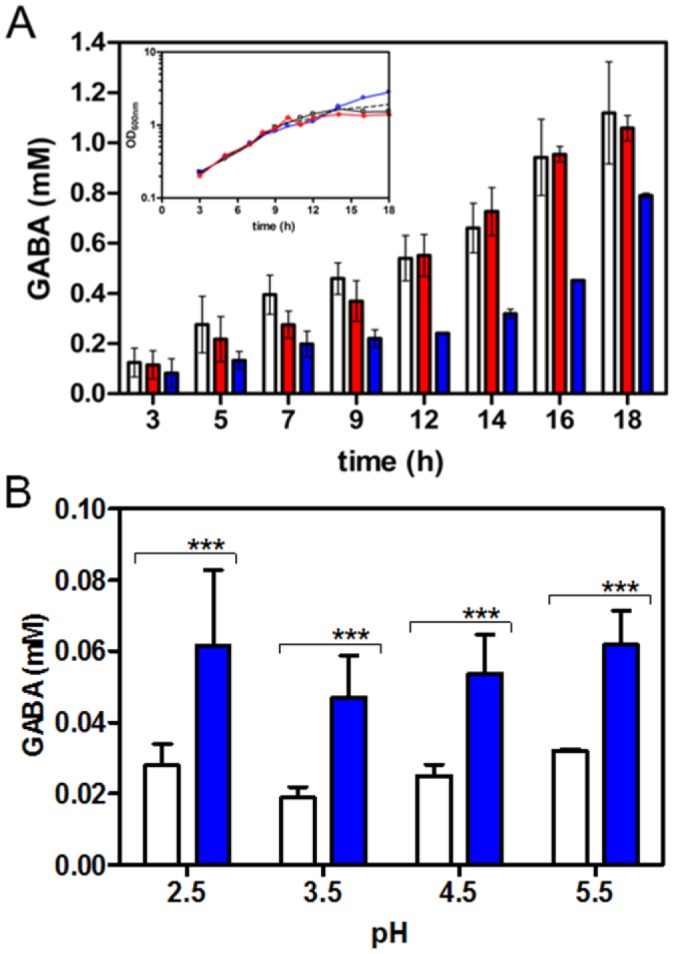
GABA export in the *E. coli* *yhiM* mutant and overexpressing strains. **(A)** The *E. coli* strains MC4100 (white) and its knockout mutants MC4100*yhiM* (blue) and MC4100*gadC* (red) were grown in minimal medium EG, pH 5.0, supplemented with 10 mM sodium glutamate. At the indicated time intervals 1-ml aliquots were collected, the OD_600_ was measured and GABA in the spent medium quantified. The values are means ± standard deviation of at least 3 independent cultures. Inset: the growth profiles (OD_600_) of MC4100 (black), MC4100*yhiM* (blue) and MC4100*gadC* (red) are reported. **(B)** Cells from IPTG-induced cultures of the *E. coli* strains JM109/pREP4/pQE60 (white) and JM109/pREP4/pQE*yhiM*-6×His (blue) were incubated at 37 °C for 90 min in EG medium, at the indicated pH, supplemented with 1 mM sodium glutamate. The GABA exported was assayed as previously described [37]. The values are means ± standard deviation of 3 independent experiments (in duplicate) with p-value of 0.0005 (***).

It has been reported that *yhiM* is amongst the genes significantly up-regulated in an *E. coli* *cydAB* mutant, in which the terminal oxidase cytochrome *bd-I*, typically induced under microaerobic conditions, is missing [19]. Based on experimental evidence, the authors suggest that GadC might substitute for terminal oxidase-mediated (cytochrome *bd-I* and *bo'*) proton translocation, thus contributing to proton motive force (PMF) maintenance. Therefore the possibility exists that *yhiM* codes for a membrane protein involved in GABA export at pH ≥ 5, where *gadC* is expected to be inactive [43]. In addition to this the localization of the *E. coli* *yhiM* homolog in *C. perfringens* in between the *gadB* and *gadC* genes (Figure 1A) suggests that YhiM might be involved in GABA transport. In order to test this possibility, we measured the amount of GABA exported by wild type *E. coli* MC4100 and by its Δ*yhiM* and Δ*gadC* isogenic derivatives grown in minimal medium EG, pH 5.0, supplemented with 10 mM glutamate. Aliquots of the growth medium were withdrawn at time intervals and assayed for GABA content. During growth the Δ*yhiM* strain exports in the extracellular medium significantly less GABA (50% on average) than the wild-type and Δ*gadC* strains, with the latter mutant strain showing no effect on GABA export under the conditions used for the assay (Figure 5A), in line with a role of GadC more restricted to pH values ≤ 3 [43]. Though less pronounced, similar results were obtained when growth was carried out in rich LBG medium (LB, pH 5.0, containing 0.4% glucose) or using *E. coli* MG1655 and its *yhiM* and *gadC* deletion mutants (data not shown).

In order to obtain further evidence on the involvement of YhiM in GABA export, we compared the extracellular GABA produced by the *E. coli* strain JM109/pREP4/pQE*yhiM*-6×His with that produced by the strain JM109/pREP4 carrying the empty expression vector pQE60. The ability to export GABA was assayed by incubating at 37 °C for 90 min a fixed amount of cells (4–5 × 10^7^) in 0.5 ml of EG medium, at different pHs, containing 1 mM glutamate. The results shown in Figure 4B confirm that YhiM is involved in GABA export and that its activity is not significantly affected by the pH of the medium. Together these results suggest that YhiM contributes to GABA export in *E. coli*.

## 4. Conclusions

In the present work we have analysed in further detail the transcriptional regulation of *yhiM* and its function in *E. coli*. Our data suggest that YhiM is dispensable for GDAR and thus its function does not overlap that of GadC, which remains a key structural component of AR under both fermentative and oxidative conditions. However, while the GABA export activity of GadC is restricted to extremely acidic pH conditions [43], as confirmed by the unaffected ability to export GABA in minimal medium at pH 5.0 (Figure 5A) of the otherwise acid-sensitive *gadC* mutant (Figure 4). As a matter of fact, the *yhiM* mutant is not impaired in GDAR (or in glutamine-dependent AR), though it exports less GABA than the parental strain in minimal medium EG at pH 5.0. Thus, we conclude that YhiM has an involvement in GABA export, though we noticed some differences among the strains under analysis (i.e. between MC4100 and MG1655). Heterogeniety at the strain level, might also provide an explanation on why we could not obtain results in agreement with those obtained by other authors, using a different *E. coli* strain and a different approach for mutagenesis (tn*10* insertion) [27].

While *yhiM* expression and function are not strictly related to AR, they might be linked to other physiological functions which also the GDAR genes participate to. Amongst these functions there are the adaptations of *E. coli* cells to respiratory stress and anaerobiosis, conditions in which many GDAR genes as well as *yhiM* are induced (Figure 1A and Table 3). The role played by YhiM is still elusive in the *E. coli* physiology and will requires further work to be clearly assessed, however the high similarity (> 60%) in amino acid sequence amongst YhiM homologs in bacteria from different phyla (See Supplementary Figure 1) reinforces the hypothesis that this protein might play an important role in bacterial cell physiology. As a matter of fact, a recent report suggests an involvement of YhiM at high temperatures and low osmolarity [44].
