# Supplementary material for: The yhiM gene codes for an inner membrane protein involved in GABA export in Escherichia coli
Source: AIMS Microbiol. 2017 Feb 17;3(1):71–87. doi: 10.3934/microbiol.2017.1.71 (PMC6604978; doi:10.3934/microbiol.2017.1.71)
Supplement: Supplementary file 1 [file microbiol-03-01-071-s001.pdf]

AIMS Microbiology

AIMS Microbiology, 3(1): 71-87.
DOI: 10.3934/microbiol.2017.1.71
Received: 01 January 2017
Accepted: 08 February 2017
Published: 17 February 2017

http://www.aimspress.com/journal/microbiology

Research article

The *yhiM* gene codes for an inner membrane protein involved in GABA export in *Escherichia coli*

Angela Tramonti [1], Fiorenzo De Santis [2], Eugenia Pennacchietti [2] and Daniela De Biase [2,]*

[1] Institute of Molecular Biology and Pathology, CNR, Department of Biochemical Sciences "A. Rossi Fanelli", Sapienza University of Rome, Piazzale Aldo Moro 5, 00185 Roma, Italy
[2] Department of medico-surgical Sciences and Biotechnologies, Sapienza University of Rome, Laboratory affiliated to Istituto Pasteur Italia-Fondazione Cenci Bolognetti, Corso della Repubblica 79, 04100 Latina, Italy

* **Correspondence:** Email: daniela.debiase@uniroma1.it; Tel: +39-0773-1757230;
Fax: +39-0773-1757254.

```
                                      1                                  2
E. coli             MNIYIGWLFKLIPLIMGLICIALG--GFVLESSGQSEYFVAGHVLISLAAICLALFTTAF 58
B. avium            MNYGISVLFRLIPLVMGVICLCLG--LLVLRDSTNPGGYTAGHVLISLTAICYALFTTAS 58
L. hongkongensis    MNYSISVLFRLIPLAMGAICLGLG--LYVLDGPPDANHFVAGHVLVSLAAICFALFTTAA 58
C. perfringens      MNYYISILFRAIPLLMGIICLTYGSYIYTFGDKFGNGYIIASHVIIFFTAICIALFTTAA 60
B. tethaiotaomicron MNYGISILFRAIPLTMAVFCFGYG--AFIYGYGDAGNRLVAGPVVFSLGMICIALFCTAA 58
P. distasonis       MNYGISVLFRAIPLLMALFCFGYG--FFVFNYGVDSSRFVAGPVVFSLGFICIALFATAA 58
                    **  *. **: *** *. :*:  *                *. *:. :  ** *** **
                                              3
E. coli             IIISQLTRGVNTFYNTLFPIIGYAGSIITMIWGWALLAGNDVMADEFVAGHVIFGVGMIA 118
B. avium            VIILQLLGNFNRSWLFLLPFSGYALAALTMGWGLSITS-IPRPADAFVSGHVVFGVGMIA 117
L. hongkongensis    TIIRQLTKTYNTFWLVMLPLLGYIVGLLTIVWGLDIIARGDLPP-YVVAGHVVFGVGLIT 117
C. perfringens      TIIRQLINKYNSFFKFILPTIGYLCAAISMLGGLYFALNNGGNPAYFVAGNIGFGVGLIA 120
B. tethaiotaomicron TIIRQIIHTYNEATKYVLPVVGYLAAIITIVGGICIFNAATTTS-AFVAGHVITGVGFIT 117
P. distasonis       TIIRQIIHTYDNVARFALPILGYLSATITFLAGFILLMSSPAPA-DFVAGHVICGVGLIT 117
                     ** *:    :      :*  **  . :::  *  :       .  .*:*::  ***:*:
                        4                                        5
E. coli             ACVSTVAASSGHFLLIPKNAAGSKSDGTPVQAYSSLIGNCLIAVPVLLTLLGFIWSITLL 178
B. avium            ACVATVAAASVRFTLIPKNSAGTHASGVPAAAYSRGAALIILMAVPLVCTLIGAIWGGSLL 177
L. hongkongensis    LCVTTVAASSSRFTLIPLNSGRPDGEPGAPGAYPASVGWLLIAVPVVCSLTGYVWALGLL 177
C. perfringens      CCVSTVALSSTKFLLIPSNSKNLKEGEKPKGCFSKEMSYVLIAIPIICTLIGLIFSFNLL 180
B. tethaiotaomicron ACVATAATSSTRFSLIPANAK-ATGNEVPEGAFSIGQRRAMIILAIVISCIAWIWAFILL 176
P. distasonis       ACVATTATASTRFTLIQMNAK-SDDPRIPDKAFNFWQGLFLILVASLISIVAWIWAYRLL 176
                     **:*.* :* :* **  *:       .  .:       :: :. : :  . ::.  **
                                           6                                7
E. coli             RS-ADITPHYVAGHVLLGLTAICACLIGLVATIVHQTRNTFSTKEHWLWCYWVIFLGSIT 237
B. avium            AD-QDTMENVVAGHVLIGIAMVCASLIALVATVARQTRNLFDEKERWYWTAWVLVMGTAA 236
L. hongkongensis    ARGADDPGFYVAGHVLFGLSTICTCLIALVATVVRQVRNQYRKSERSFWIWLVIVLGLAD 237
C. perfringens      RH-TDKVPSLVAGFVLFGISLVCGSLISLVASVCRQIQNTFTNKERWKWSILVIVLGSIN 239
B. tethaiotaomicron SNSHSHPAYFVAGHVMVGLACICTSLIALVATIARQVRNDYSERERNKWPKLVLLMGSIS 236
P. distasonis       AHSEEHSQYFVAGHVMAGLACICTSLIALVATIARQIRNTYSRLEKRLWHRFVILMGSIS 236
                        .     ***.*: *:: :* .**.***:: :* :* :   *:  *   *:.:*
                                              8
E. coli             VLQGIYVLVSSDASARLAPGIILICLGMICYSIFSKVWLLALVWRRTCSLANRIPMIPVF 297
B. avium            LLLGIYVLSTGTQGYRLAPGIVLVGLGMICYSIVSKVLLLALVWRRESKLANRVPLIPVL 296
L. hongkongensis    IIFGIAVLVDSPASFHVAPGLILIGIGMICFSILSKVLLLGLVWRARFALANRVPILPVM 297
C. perfringens      FLLGICVLLFSKNQTFIAPGYVLIGLGLVCYSISSKVLLLASVWRRKSKLSKRIPLIPVI 299
B. tethaiotaomicron FVWGIFVILADSGSANGTTGYIMLGLGLVCYSISSKVILLAKIWRREFKLANRIPMIPVL 296
P. distasonis       LIWGLFVLGDSDP-ANASTGYIMIGLGLVCYSISSKVILLSKIWREEFKLANRIPLIPIF 295
                    .: *: *:         :.* ::: :*::*:** *** **. :**    *::*:*::*::
                        9                                 10
E. coli             TCLFCLFLASFLAEMAQTDMGYFIPSRVLVGLGAVCFTLFSIVSILEAGSAKK-- 350
B. avium            TCLACLFCAAFLAEANVTDPNYFIASRILTGLGAVCFTLFSIVSILEAGTSKKAA 351
L. hongkongensis    TALACLFLSAFVFEAGDLSAAYFIPARVLVGLGAVCFTLFSIVSILEAGTSGD-- 350
C. perfringens      TALACLFLSAYLSEAELVNMAYFVPAHVMVGLGAICFTLFSIVSILESGTSN--- 351
B. tethaiotaomicron TALACLFLAAFVFELATVNADYFIPARVLVGLGAICFTLFSIVSILESGTSSK-- 349
P. distasonis       TALFCLFLSAFLFEMAAEHSYYAIPARVLAGLGAICFTLFSIVSILESGTSSK-- 348
                    *.* *** :::: *       * :.:::.****:************:*::
```

Supplementary Figure 1. Multiple sequence alignment of *E. coli* YhiM with its orthologs in other bacterial species. The sequences shown are from: *E. coli* MG1655 YhiM [Swiss-Prot: P37630], *Bordetella avium* 197N [Swiss-Prot: Q2KZQ9], *Laribacter hongkongensis* HLHK9 [Swiss-Prot: C1D8T3], *Clostridium perfringens* str.13 [Swiss-Prot: Q8XIQ5], *Bacteroides thetaiotaomicron* VPI-5482 [Swiss-Prot: Q8A9L0], and *Parabacteroides distasonis* ATCC 8503 [Swiss-Prot: A6L8F3]. The horizontal lines are above the residues predicted by TMHMM to form the TM domains in *E. coli* YhiM. Below the alignment asterisks, colons and dots are for identical, highly conserved and conserved amino acids, respectively.
